# Alpha-mangostin induces endoplasmic reticulum stress and autophagy which count against fatty acid synthase inhibition mediated apoptosis in human breast cancer cells

**DOI:** 10.1186/s12935-019-0869-z

**Published:** 2019-05-31

**Authors:** Wenyuan Huang, Yan Liang, Xiaofeng Ma

**Affiliations:** 1 College of Life Sciences, University of Chinese Academy of Sciences, No. 19A Yuquan Road, Beijing, 100049 China; 2 School of Kinesiology and Health, Capital University of Physical Education and Sports, No. 11 Beisanhuanxi Road, Beijing, 100191 China

**Keywords:** Fatty acid synthase, Alpha-mangostin, Inhibitor, Autophagy, Endoplasmic reticulum stress

## Abstract

**Background/aims:**

One of the most important metabolic hallmarks of breast cancer cells is enhanced lipogenesis. Increasing evidences suggest that fatty acid synthase (FAS) plays an important role in human breast cancer. Previously we discovered that alpha-mangostin showed apoptotic effect on human breast cancer cells via inhibiting FAS activity. The endoplasmic reticulum (ER) stress and autophagy are involved in cell apoptosis. However, the role of ER stress and autophagy in FAS inhibition induced apoptosis still remains unclear.

**Methods:**

We evaluated the effects of alpha-mangostin on ER stress and autophagy in human breast cancer cells. Intracellular FAS activity was measured by a spectrophotometer at 340 nm of NADPH absorption. Cell Counting Kit assay was used to test the cell viability. Immunoblot analysis was performed to detect protein expression levels. Apoptotic effects were detected by flow cytometry.

**Results:**

Alpha-mangostin induced endoplasmic reticulum stress and autophagy, both of which reduced the apoptotic effect of alpha-mangostin in MDA-MB-231 cells. Palmitic acid, the end product of FAS catalyzed reaction, rescued the ER stress and autophagy induced by alpha-mangostin. Cell apoptosis was markedly promoted by inhibiting ER stress and autophagy while treating cells with alpha-mangostin.

**Conclusion:**

We propose a hypothesis that a combination of FAS inhibition and ER stress and autophagy inhibition has an application potential in the chemoprevention and treatment of breast cancer.

**Electronic supplementary material:**

The online version of this article (10.1186/s12935-019-0869-z) contains supplementary material, which is available to authorized users.

## Background

Breast cancer remains one of the most common human cancers and the second leading cause of cancer mortality in women worldwide [1]. As in other cancers, elevated lipogenesis is one of the most important metabolic hallmarks of breast cancer cells [2]. Cancer cells acquire fatty acids mainly through de novo lipogenesis, in spite of sufficient dietary lipid supply, to support their growth and proliferation [3–5]. The upregulated fatty acid synthesis in cancer cells is reflected by significant increase in both expression and activity of fatty acid synthase (FAS, EC 2.1.3.85) [6]. FAS catalyzes NADPH-dependent condensation of acetyl-coenzyme A (CoA) and malonyl-CoA to produce palmitate [7]. Both FAS activity and expression level are increased in oncogenesis during cancer progression, and FAS-overexpressing cancers exhibit more aggressive phenotypes [8]. This cancer-specific elevation of FAS-dependent lipogenesis, however of minor importance in normal cells, would render cancer cells more vulnerable to anti-cancer drugs targeting FAS [8, 9].

The endoplasmic reticulum (ER) is a place where proteins are modified in eukaryocyte cytoplasm and is the major site of lipid metabolism, as many enzymes involved in lipid metabolism are located in the ER [10]. ER homeostasis is destroyed by accumulating misfolded or unfold proteins in the ER lumen. To cope with the changes of extracellular environment and recover ER surroundings, the ER stress is induced. The unfold protein response (UPR) is a collection of signaling pathways that is activated to overcome the ER stress [11, 12]. Three ER transmembrane receptors, protein kinase R-like endoplasmic reticulum kinase (PERK), inositol-requiring enzyme 1 (IRE1), and activating transcription factor 6 (ATF6), initiate UPR through a signaling network [13]. When UPR fails to rebuild homeostasis, programmed cell death could be induced to eliminate injured cells [13]. In short ER stress is a protection for cells to cope with unfriendly environment. However, prolonged ER stress and activation of UPR pathways can lead to apoptosis and autophagy.

Autophagy plays a critical role in maintaining the intercellular homeostasis under both physiological and pathological conditions [14, 15]. Autophagy could be activated as an adaptive process responding to metabolic stress and UPR that eventually results in a possible compensatory reaction to relieve the burden of unfolded proteins and damaged organelles by autophagic degradation [16]. In recent years many evidences show autophagy plays an important role in cancer growth and progression [17]. The role of natural product in the induction of autophagy has also been investigated [18]. However, autophagy participates in both cell death and cell survival [19].

Alpha-mangostin (α-mangostin) is the most abundant xanthone exists in mangosteen pericarp and has been confirmed to have anti-proliferative and apoptotic effects in various types of human cancer cells [20–23]. Our previous study showed that α-mangostin induced breast cancer cells apoptosis via inhibiting intracellular FAS activity [24, 25]. However, the specific mechanism involved still needs to be clarified. Because ER stress and autophagy have been involved in the induction of cancer cell apoptosis [26, 27], we questioned whether they are implicated in α-mangostin induced breast cancer cells apoptosis. Moreover, we questioned what the role of ER stress and autophagy is in α-mangostin induced apoptosis.

In the present study the effects of α-mangostin on FAS inhibition, ER stress, autophagy, as well as the apoptosis in human breast cancer MDA-MB-231 and MCF-7 cells were investigated. We found that α-mangostin induced ER stress and autophagy, both of which were benefit for cell survival. The inhibition of ER stress or autophagy enhanced α-mangostin induced apoptosis significantly. These results suggested that inhibition of FAS combined with ER stress and/or autophagy inhibition might provide new clues into breast cancer treatment.

## Methods

### Reagents

Acetyl-CoA, Malonyl-CoA, NADPH, DMSO, 3-methyladenine (3MA), 4-phenylbutyric acid (4PBA), and α-mangostin were purchased from Sigma (St. Louis, MO, USA). Dulbecco’s Modified Eagle’s Medium (DMEM) was purchased from Gibco (Beijing, China). Fetal bovine serum (FBS) was purchased from Every Green (Zhejiang, China). Antibodies of FAS, PARP, P62 and GAPDH were purchased from Cell Signaling Technology (Denvers, MA, USA). Antibodies of BIP and CHOP were purchased from Proteintech (Rosemont, IL, USA). Antibody of LC3B was purchased from Abcam (Cambridge, MA, USA). Antibodies of ATF6, PERK, and IRE1 were purchased from Cohesion (Maidenhead, Berkshire, UK).

### Cell lines and culture

The human breast epithelial cell lines MDA-MB-231, triple negative breast cancer cells derived from a metastatic carcinoma, and MCF-7, estrogen receptor-positive cells derived from an in situ carcinoma, were used in the study. Cells were purchased from the Type Culture Collection of the Chinese Academy of Sciences, Shanghai, China. The cells were grown in DMEM supplemented with 10% fetal bovine serum. Cells were maintained at 37 °C in a humidified atmosphere containing 5% CO_2_.

### Cell viability assay

Cell viability was assessed by a Cell Counting Kit (CCK-8; Dojindo Laboratories, Kumamoto, Japan). Cells were seeded at a concentration of 1 × 10^6^ cells/mL into 96-well plates, and were allowed an overnight period for attachment. The medium was removed and fresh medium was added along with the drugs. Following treatment, a drug-free medium (100 μL/well) and 10 μL CCK-8 solution were added into cells, which were then incubated for 1 h at 37 °C. The optical density (OD) value (absorbance) was measured at 450 nm by a microplate spectrophotometer (Multiskan, MK3). All experiments were performed in quadruple on three separate occasions.

### Intracellular free fatty acid quantify assay

Intracellular free fatty acid was measured according to the previous description [25]. Briefly, after treatment with α-mangostin for 24 h, cells were harvested using trypsin–EDTA, washed twice with phosphate buffer saline (PBS). Intracellular fatty acid was determined with a Free Fatty Acid Quantification Kit (Bivision) according to the manufacturer’s instructions.

### Detection of cell apoptotic rates by flow cytometry

Cell apoptosis detection was performed using an Annexin-VFITC Apoptosis Detection Kit (BD company, US) according to the manufacturer’s protocol. Briefly, cells were collected after 24 h treatment with drug. The cells were washed twice with cold PBS then resuspended in 1× binding buffer at a concentration of 1 × 10^6^ cells/mL. Then 500 μL of the cell suspension was incubated with 5 μL annexin-V-FITC and 10 μL PI for 15 min in the dark and analyzed by a FACScalibur instrument (Becton–Dickinson, San Jose, US) within 1 h. Apoptotic cells are those stained with annexin V+/PI− (early apoptotic) plus annexin V+/PI+ (late apoptotic cell).

### Western blot analysis

Cells were washed three times with ice-cold PBS and harvested in RIPA lysis buffer with 1 mM PMSF, and then lysed on ice for 10 min. The homogenate was centrifuged at 13,800*g* for 15 min at 4 °C and supernatant was collected for subsequent analysis. Equal protein extracts were separated by 10% SDS-PAGE. Then electrophoretically transferred to PVDF membranes for 2 h. Then membranes were blocked with 5% skimmed milk for 1–2 h at room temperature to prevent nonspecific antibody binding, and probed with various primary antibodies dilution at a concentration of 1:1000 recommended by the suppliers overnight at 4 °C. Then washed three times with TBST (10 mM Tris, 10 mM NaCl, 0.1% Tween 20), and incubated 1 h with corresponding secondary antibody at a concentration of 1:10,000 and developed with a commercial kit (West Pico chemiluminescent substrate). Blots were reprobed with an antibody against GAPDH as the control of protein loading and transfer. The density of the bands was measured by Image Lab. All experiments were performed three times.

### Monodansylcadaverine staining

Autophagosome was stained by monodansylcadaverine (MDC) according to the manufacturer’s instructions. Cells were seeded at a concentration of 1 × 10^6^ cells/mL into 24-well plates, cultured overnight and changed fresh DMEM contained 0, 1, 2, 4 μM α-mangostin. After 24 h treatment with α-mangostin, cells were washed three times with PBS, then add 100 μL washing buffer within 10 μL MDC Strain. After 30 min staining in dark, cells were washed twice and added 100 μL Collection Buffer. Observation by fluorescence microscope with λex = 355 nm/λem = 512 nm, images were captured using ImagePro Plus software.

### Detection of the mitochondrial membrane potential

The mitochondrial membrane potential (ΔΨm) was detected by mitochondrial membrane potential kit with JC-1 (Beyotime Biotechnology, China) under the manufacturer’s protocol. JC-1 is a cationic dye that accumulates in energized mitochondria. In healthy mitochondria, due to high mitochondrial membrane potential, JC-1 aggregates yielding a red fluorescence. On the other hand, in dysfunctional mitochondria with low mitochondrial membrane potential, JC-1 is predominantly a monomer that yields green fluorescence. MDA-MB-231 cells were seeded into 6-well plates with a concentration of 1 × 10^6^ cells/mL and then treated with different concentrations of α-mangostin (0, 1, 2, 4 µM). After 24 h, cells were digested by trypsin, resuspended in 1× binding buffer at a concentration of 1 × 10^6^ cells/mL and then were washed three times with cold PBS. After that, cells were stained with 25 µM JC-1 and incubated at 37 °C for 30 min. Finally, cells were analyzed with a flow cytometer. The fluorescence shift of JC-1 from red to green was measured with the green channel (488 nm/525 nm) to assess the mitochondrial dysfunction.

### RNA interference

The P62-targeted siRNA (GGAGUCGGAUAACUGUUCATT) and the negative RNA (UUCUCCGAACGUGUCACGUTT) were purchased from GenePharma (Shanghai China). The siRNA was transfected into cells with the use of Lipofectamine 2000 reagent (Invitrogen) according to the manufacturer’s instructions. At 24 h after transfection, cells were used for analyses of P62 expression by western blot.

### Intracellular FAS activity assay

After a residence time of 24 h exposure to α-mangostin, cells were harvested by trypsinization, pelleted by centrifugation, washed twice, and resuspended in cold PBS. Cells were sonicated at 4 °C and centrifuged at 13,800*g* for 15 min at 4 °C to obtain particle-free supernatants. The FAS activity was determined spectrophotometrically by measuring the decrease of absorbance at 340 nm due to oxidation of NADPH as others previously described [25]. 50 µL particle-free supernatant, 25 mM KH_2_PO_4_–K_2_HPO_4_ buffer, 0.25 mM EDTA, 0.25 mM dithiothreitol, 30 µM acetyl-CoA, 350 µM NADPH (pH 7.0) in a total volume of 500 µL were monitored at 340 nm for 60 s to measure background NADPH oxidation. After the addition of 100 mM malonyl-CoA, the reaction was assayed for an additional 60 s to determine the FAS dependent oxidation of NADPH.

### Statistical analysis

Data represent the mean ± standard deviation (SD) from at least three independent experiments. The unpaired Student’s t test was used to compare the means of two groups. The statistical differences among three or more groups were determined by one-way ANOVA with Tukey’s post-test using Origin8.5 software (Originlab, Northampton, MS, USA). Statistical significance was determined at the level of p < 0.05.

## Results

### α-Mangostin stimulated autophagy in human breast cancer cells

**Fig. 1:**
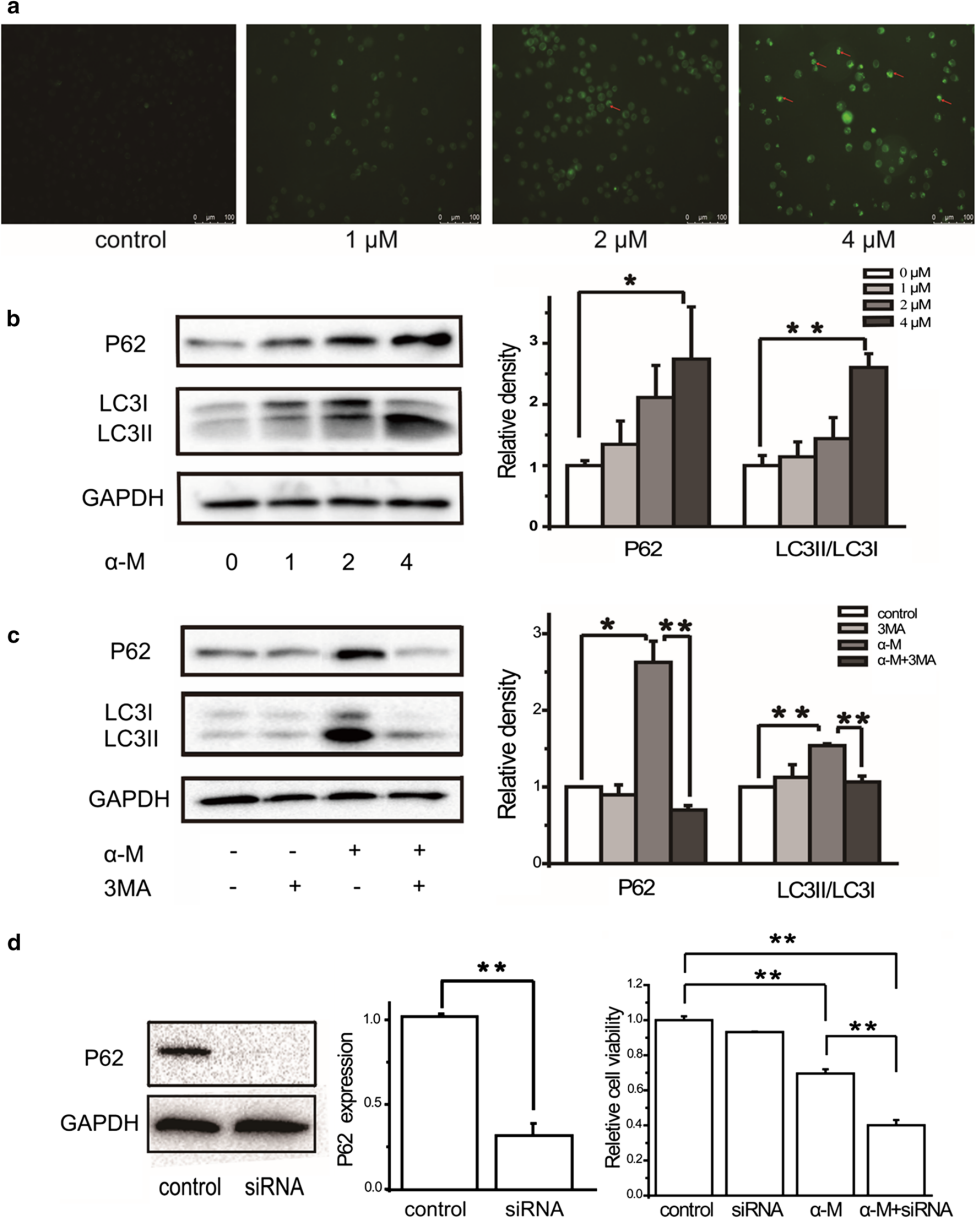
α-Mangostin stimulated autophagy in MBA-MB-231 cells. **a** MDA-MB-231 cells were treated with 0, 1, 2, and 4 μM α-mangostin for 24 h, then strained by MDC. **b** MDA-MB-231 cells were treated with 0, 1, 2, and μM α-mangostin for 24 h, and then the relative expression levels of LC3II/LC31 and P62 were analyzed by western blot and were quantified densitometrically with the software ImageJ and calculated according to the reference bands of GAPDH. Data represented the mean ± SD of three independent experiments. *p < 0.05, **p < 0.01. **c** Cells were treated with/without 4 μm α-mangostin followed 24 h incubation with/without 3MA and the relative expression levels of proteins were analyzed and quantified. Data represented the mean ± SD of three independent experiments. *p < 0.05, **p < 0.01. **d** MDA-MB-231 cells were treated with/without 4 μm α-mangostin with/without transfected with siRNA targeting P62 for 24 h. Cell viabilities were then determined by the CCK-8 assay. Data represented the mean ± SD of three independent experiments. *p < 0.05, **p < 0.01. α-M: α-mangostin

**Fig. 2:**
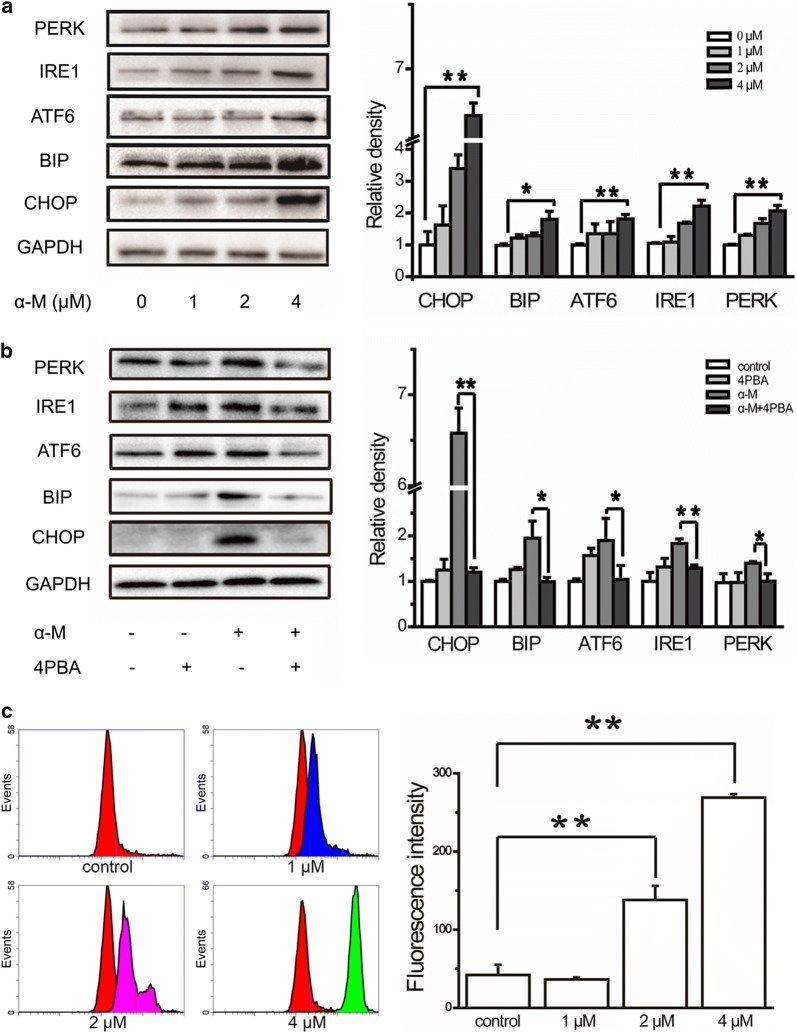
α-Mangostin induced ER stress in MBA-MB-231 cells. **a** MDA-MB-231 cells were treated with 0, 1, 2, and 4 μM α-mangostin for 24 h, and then the relative expression levels of CHOP, BIP, ATF6, IRE1, and PERK were analyzed by western blot and were quantified densitometrically with the software ImageJ and calculated according to the reference bands of GAPDH. Data represented the mean ± SD of three independent experiments. *p < 0.05, **p < 0.01. **b** The relative expression levels of CHOP, BIP, ATF6, IRE1 and PERK in cells treated with/without 4 μm α-mangostin followed 24 h incubation with/without 4PBA were analyzed by western blot and quantified. Data represented the mean ± SD of three independent experiments. *p < 0.05, **p < 0.01. **c** Cells were treated with different concentrations of α-mangostin (0, 1, 2, and 4 μM). After 24 h, cells were stained with JC-1 and analyzed by flow cytometry. Histogram showed cells treated with DMSO alone (red), 1 μM α-mangostin (blue), 2 μM α-mangostin (pink) and 4 μM α-mangostin (green). For comparison, the control group (red histogram) was merged into every histogram. The corresponding bar graph showed the mean green fluorescence intensity, which was measured with excitation/emission: 488/525 nm by flow cytometer. Data represented the mean ± SD of three independent experiments. **p < 0.01

To identify whether α-mangostin triggered autophagy, a series of experiments were performed. Firstly, MDC, an auto-fluorescent dye which can accumulate in the acidic vesicular organelles, was used to detect autophagic activity. MDA-MB-231 cells were treated with 0, 1, 2, and 4 μM α-mangostin for 24 h, strained by MDC, and observed by Fluorescence Microscopy. As shown in Fig. 1a, 2 and 4 μM α-mangostin induced the formation of autophagic cytoplasmic vesicles in MDA-MB-231 cells (autophagosomes), suggesting the occurrence of a state of increased intracellular autophagy in MDA-MB-231 cells.

LC3II/LC3I and P62 were the marker of autophagy, which played important roles in formation of the autophagosome. The expression levels of LC3II/LC3I and P62 in α-mangostin treated MDA-MB-231 cells and MCF-7 cells were detected. Western blot results showed that after treating with α-mangostin, the ratio of LC3II/LC3I was increased significantly, meanwhile the expression level of P62 was also significantly promoted in a dose-dependent manner, indicating that α-mangostin facilitated the autophagy level in MDA-MB-231 cells and MCF-7 cells, as is shown in Fig. 1b and Additional file 1: Figure S1.

In addition, we measured the effects of 3MA, an autophagy inhibitor, on α-mangostin treated cells. As shown in Fig. 1c, the expression levels of LC3II/LC3I and P62 did not change when the cells were treated with 5 mM 3MA alone. However, compared with α-mangostin treatment alone, a combine administration of both α-mangostin (4 μM) and 3MA (5 mM) resulted in a significant decrease of P62 and LC3II/LC3I ratio. All the above results confirmed that α-mangostin did stimulate autophagy in MDA-MB-231 cells.

A knockdown study using siRNA of P62 with the treatment of α-mangostin was also performed. To specifically silence the expression of P62, MDA-MB-231 cells were transfected with siRNA-targeting P62 as described in Methods. As shown Fig. 1d, P62 RNAi severely suppressed the expression level of this protein. Cell viability results showed that knockdown of P62 did not affect the cell viability, however, a combination treatment of both α-mangostin and P62 RNAi showed stronger cell toxicity.

### α-Mangostin induced ER stress in human breast cancer cells

To test whether α-mangostin induced ER stress in MBA-MB-231 cells, the protein levels of CHOP, BIP, and the UPR markers (IRE1, PERK, ATF6) were determined by western blot. As shown in Fig. 2a, α-mangostin administration significantly up-regulated the expression levels of IRE1, ATF6, PERK, CHOP, and BIP. The expression levels of both CHOP and BIP were up-regulated in MCF-7 cells (Additional file 1: Figure S1). These results proved that α-mangostin caused ER stress in human breast cancer cells. The results of the time course study showed that the expression level of CHOP in MDA-MB-231 cells was up-regulated in 6 h. However, the expression level of BIP was up-regulated in 12 h, as shown in Additional file 2: Figure S2. The effects of 4PBA, an ER stress inhibitor, on α-mangostin treated and untreated cells were investigated. The results showed that compared with α-mangostin treatment alone, a combination of 4PBA (5 mM) and α-mangostin (4 μM) down-regulated the expression levels of ER stress related proteins. As a comparison, 4PBA treatment alone did not affect the expression levels of these proteins. These results suggested that α-mangostin induced ER stress which could be inhibited by 4PBA (as shown in Fig. 2b). ER stress markers alteration is one of the ways to show mitochondrial stress [28]. In order to detect the effect of α-mangostin on mitochondria, the mitochondrial membrane potential after α-mangostin treatment was measured. As shown in Fig. 2c, after exposed to α-mangostin (0, 1, 2, and 4 μM) for 24 h, the mean green fluorescence intensity was increased significantly, which reflected a reduction of the mitochondrial membrane potential. These results indicated that α-mangostin caused mitochondrial stress or damage.

### Combination treatment with α-mangostin and 3MA prevented ER stress

**Fig. 3:**
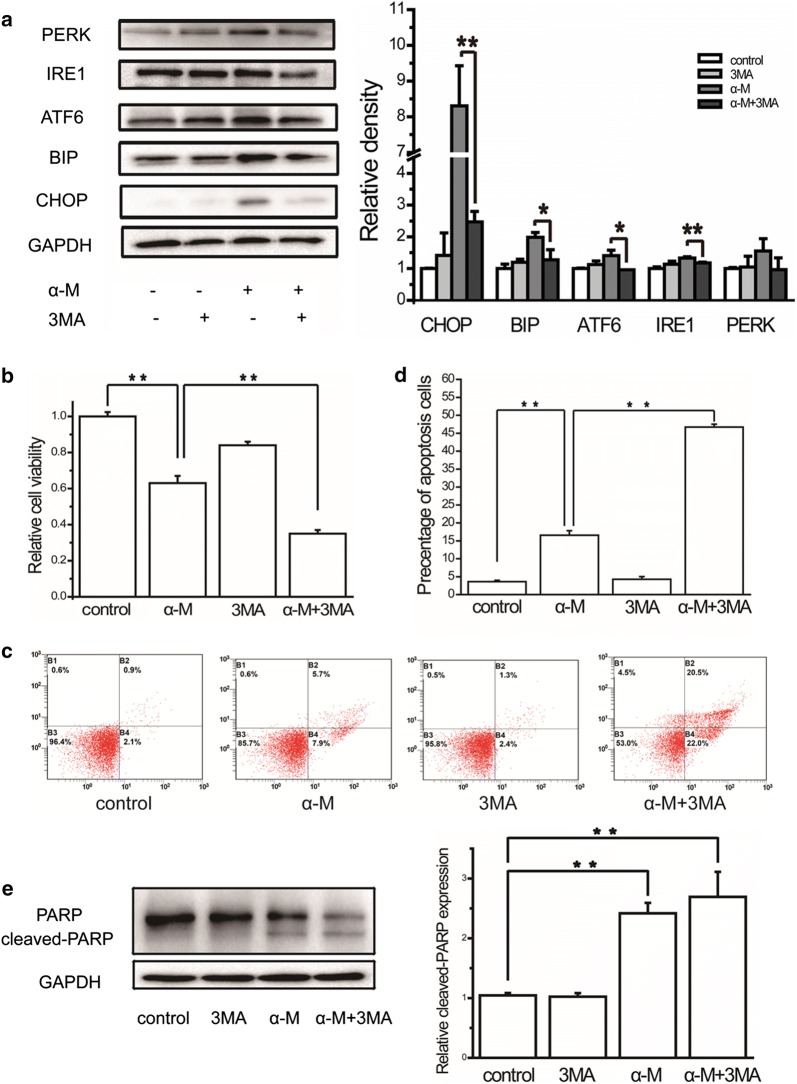
The effects of autophagy inhibitor on ER stress, cell viability and apoptosis. **a** The relative expression levels of CHOP, BIP, ATF6, IRE1 and PERK in cells treated with/without 4 μm α-mangostin followed 24 h incubation with/without 3MA were analyzed by western blot and quantified. Data represented the mean ± SD of three independent experiments. *p < 0.05, **p < 0.01. **b** Cells were treated with/without 4 μm α-mangostin followed 24 h incubation with/without 3MA. Cell viabilities were then determined by the CCK-8 assay. Data represented the mean ± SD of three independent experiments. *p < 0.05, **p < 0.01. **c** Cells were treated with/without 4 μm α-mangostin followed 24 h incubation with/without 3MA and double-stained with annexin V and PI and analyzed by flow cytometry. The gate setting distinguished between living (bottom left), necrotic (top left), early apoptotic (bottom right), and late apoptotic (top right) cells. **d** The percentage of apoptotic cells in each well was counted under flow cytometry. Data represented the mean ± SD of three independent experiments. *p < 0.05, **p < 0.01. **e** α-Mangostin and 3MA induced apoptosis in MDA-MB-231 cells as assessed by PARP cleavage. The expression levels of PARP and cleaved PARP were analyzed by western blot and were quantified densitometrically with the software ImageJ and calculated according to the reference bands of GAPDH. Data represented the mean ± SD of three independent experiments. *p < 0.05, **p < 0.01

To understand whether there was a correlation between α-mangostin induced ER stress and autophagy, we measured the effect of autophagy on ER stress. As shown in Fig. 3a, when treated MDA-MB-231 cells with α-mangostin—3MA combination, the expression levels of CHOP and BIP were significant down-regulated, meanwhile the expression levels of proteins related to three UPR pathways were also decreased. This result suggested that α-mangostin induced ER stress could be rescued by autophagy inhibition.

### Combination treatment with α-mangostin and 4PBA prevented autophagy

**Fig. 4:**
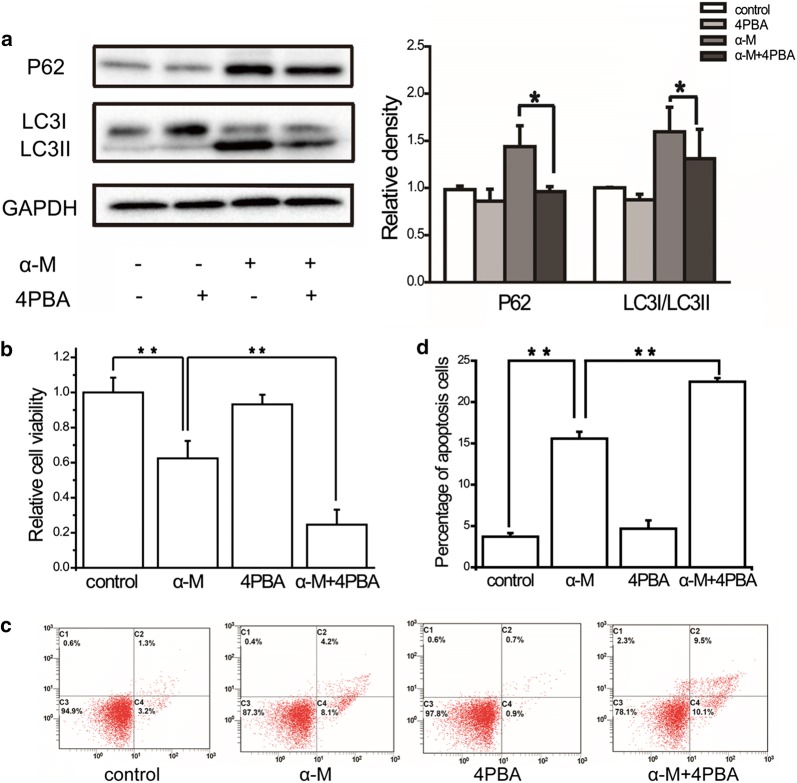
The effects of ER stress inhibitor on autophagy, cell viability and apoptosis. **a** The relative expression levels of LC3II/LC3I and P62 in cells treated with/without 4 μm α-mangostin followed 24 h incubation with/without 4PBA were analyzed by western blot and quantified. Data represented the mean ± SD of three independent experiments. *p < 0.05, **p < 0.01. **b** Cell were treated with/without 4 μm α-mangostin followed 24 h incubation with/without 4PBA. Cell viabilities were then determined by the CCK-8 assay. Data represented the mean ± SD of three independent experiments. *p < 0.05, **p < 0.01. **c** Cells were treated with/without 4 μm α-mangostin followed 24 h incubation with/without 4PBA and double-stained with annexin V and PI and analyzed by flow cytometry. The gate setting distinguished between living (bottom left), necrotic (top left), early apoptotic (bottom right), and late apoptotic (top right) cells. **d** The percentage of apoptotic cells in each well was counted under flow cytometry. Data represented the mean ± SD of three independent experiments. *p < 0.05, **p < 0.01

To further explore the relationship between autophagy and ER stress induced by α-mangostin in MDA-MB-231 cells, the effects of α-mangostin—4PBA combination were also investigated. As shown in Fig. 4a, the expression levels of LC3II/LC3I and P62 were decreased evidently. This result revealed that ER stress inhibition depressed α-mangostin induced autophagy.

### Autophagy and ER stress protected against α-mangostin induced apoptosis in breast cancer cells

**Fig. 5:**
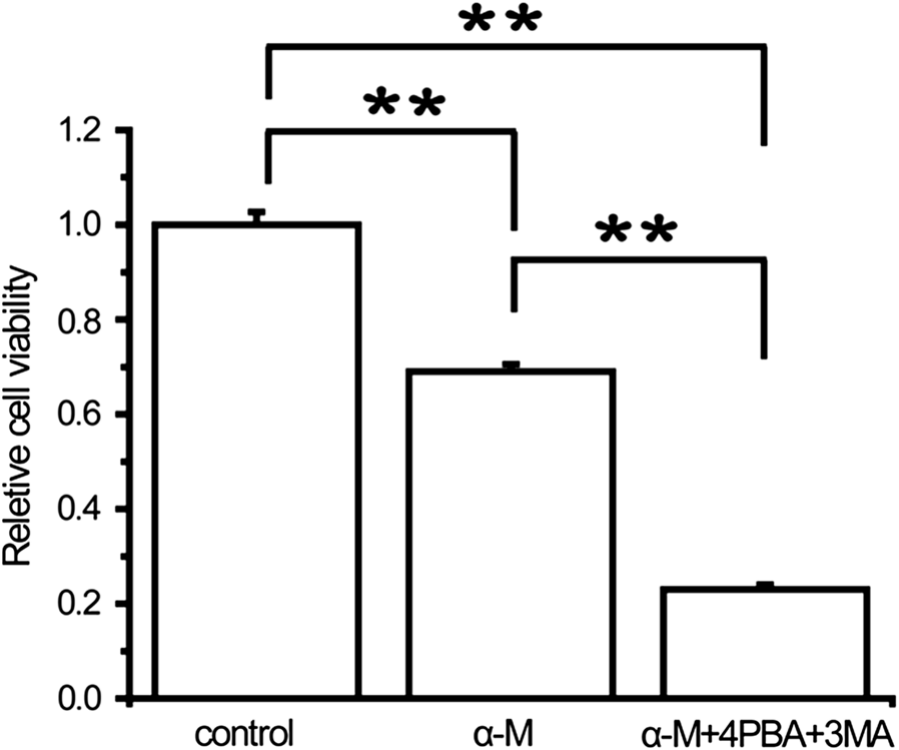
The effects of combination treatment of ER stress inhibitor, autophagy inhibitor, and α-mangostin on cell viability. Cells were treated with 4 μm α-mangostin, or a combination of 4 μm α-mangostin, 5 mM 4PBA, and 5 mM 3MA. Cell viabilities were then determined by the CCK-8 assay. Data represented the mean ± SD of three independent experiments. **p < 0.01

Having clearly established that α-mangostin activated autophagy and ER stress in MDA-MB-231 cells, we next sought to determine how autophagy and ER stress contributed to cell death. Firstly, the effects of α-mangostin—3MA combination on cell viability and apoptosis were investigated. As shown in Fig. 3b–d, compared with α-mangostin, the drugs combination reduced the cell viability and promoted apoptosis. These results showed that autophagy inhibition enhanced the apoptotic effect of α-mangostin. Secondly, to explore the biological role of ER stress in α-mangostin induced cell apoptosis, 4PBA was used to prevent ER stress. After treating with both α-mangostin and 4PBA, the viability of MDA-MB-231 cells decreased clearly compared to those cells treated with α-mangostin alone. In addition, 4PBA increased the percentage of cell apoptosis in α-mangostin treated cells (as shown in Fig. 4b–d). These results revealed that ER stress inhibition enhanced the apoptotic effect of α-mangostin. Furthermore, the combined effects of autophagy inhibitor, ER stress inhibitor, and α-mangostin on cell viability were detected. The results showed that a combination of 4PBA, 3MA, and α-mangostin caused a significant reduction of cell viability in both MDA-MB-231 and MCF-7 cells (as shown in Fig. 5 and Additional file 1: Figure S1). From these results above, we confirmed that both autophagy and ER stress played positive roles in cell survivals in α-mangostin treated cells.

### Palmitic acid (PA) recovered ER stress, autophagy, and cell apoptosis induced by α-mangostin

**Fig. 6:**
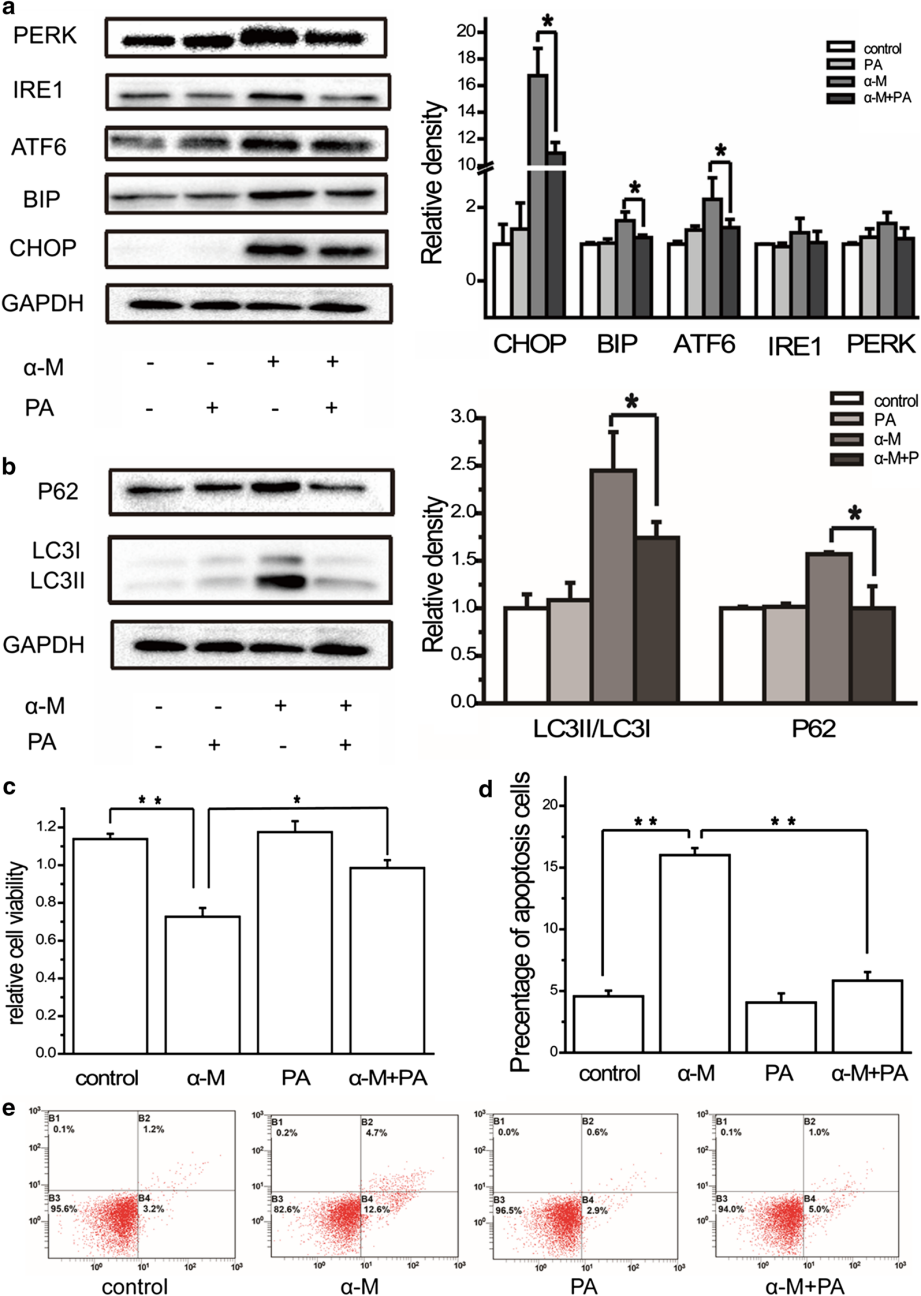
The effects of PA on ER stress, autophagy, cell viability as well as apoptosis. **a** The relative expression levels of CHOP, BIP, ATF6, IRE1 and PERK in cells treated with/without 4 μm α-mangostin followed 24 h incubation with/without 10 μM PA were analyzed by western blot and quantified. Data represented the mean ± SD of three independent experiments. *p < 0.05, **p < 0.01. **b** The relative expression levels of LC3II/LC3I and P62 in cells treated with/without 4 μm α-mangostin followed 24 h incubation with/without 10 μM PA were analyzed by western blot and quantified. Data represented the mean ± SD of three independent experiments. *p < 0.05, **p < 0.01. **c** Cells were treated with/without 4 μm α-mangostin followed 24 h incubation with/without 10 μM PA. Cell viabilities were then determined by the CCK-8 assay. Data represented the mean ± SD of three independent experiments. *p < 0.05, **p < 0.01. **d** The percentage of apoptotic cells in each well was counted under flow cytometry. Data represented the mean ± SD of three independent experiments. *p < 0.05, **p < 0.01. **e** Cells were treated with/without 4 μm α-mangostin followed 24 h incubation with/without 10 μM PA and double-stained with annexin V and PI and analyzed by flow cytometry. The gate setting distinguished between living (bottom left), necrotic (top left), early apoptotic (bottom right), and late apoptotic (top right) cells

Given that our previous experiments showed that α-mangostin inhibited intracellular FAS activity, which then reduced the amount of free fatty acids, we next queried whether the ER stress and autophagy induction effect of α-mangostin was related to its FAS inhibitory activity. To explore whether FAS inhibition was one of the reasons for α-mangostin inducing ER stress and autophagy, MDA-MB-231 cells were exposed to 4 μM α-mangostin for 24 h in the presence and the absence of 10 μM PA, the end product of FAS catalyzed reaction. As shown in Fig. 6a, α-mangostin increased the expression levels of CHOP, BIP and ATF6 significantly, and increased the expression levels of IRE1 and PERK to some extent (did not reach statistical significance). However, the combination treatment of both PA and α-mangostin reduced the expression levels of CHOP, BIP, ATF6, IRE1 and PERK. Compared with α-mangostin treatment alone, the combination treatment of both PA and α-mangostin down-regulated the expression levels of LC3II/LC3I and P62, indicating a rescue of autophagy, as shown in Fig. 6b. All these results showed that PA rescued the ER stress and autophagy induction effect of α-mangostin.

Cell viability and apoptosis results showed that PA reduced the cytotoxic effects of α-mangostin, as the cell viability was significantly increased and the percentage of apoptosis was decreased significantly after the addition of exogenous PA (Fig. 6c–e). Consistent with our previous report, these results proved FAS inhibition contributed to the cytotoxicity of α-mangostin.

## Discussion

Targeting intracellular FAS activity may represent a new approach to prevent or treat human breast cancer. α-Mangostin is a high efficiency FAS inhibitor which inhibits FAS overall reaction with a half-inhibitory concentration (IC_50_) value of 5.54 μM [24]. In our previous study, we have revealed that α-mangostin reduced viability and induced apoptosis in human breast cancer MDA-MB-231 and MCF-7 cells via both inhibiting intracellular FAS activity and down-regulating FAS expression [25]. In the present work, we demonstrated, for the first time, that α-mangostin induced both autophagy and ER stress, which were related with the FAS inhibitory effect of α-mangostin.

The reason why FAS inhibition was able to activate autophagy and ER stress in MDA-MB-231 cells is speculative. Autophagy is a process that is involved in maintaining cell viability in case of senescence or metabolic imbalance [29–31]. Under the condition of nutrient deprivation, autophagy is rapidly up-regulated and then provides an alternative source of intracellular building blocks and substrates for the generation of energy to enable cell survival [32]. Among intracellular nutrients, fatty acid plays an important role in cancer cells. Literatures collectively propose that cancer cells increase the synthesis of fatty acids to maintain growth and increase proliferation [33]. FAS is, to the best of our knowledge, the only enzyme that catalyzes the de novo synthesis of endogenous long-chain fatty acids and plays an essential role in cell division and intracellular lipid synthesis [34, 35]. The deficiency of intercellular fatty acid, caused by the inhibition of FAS, would stimulate the autophagy. In the present work, we found that when MDA-MB-231 cells were treated with α-mangostin, the intracellular FAS activity was inhibited significantly and consequently the amount of free fatty acids was reduced (Additional file 3: Figure S3). Accompany with the reduction of fatty acid, α-mangostin activated autophagy in a dose-dependent manner. This is in line with well-established evidence that cancer cells are capable of activating autophagy to survive under nutrient deficiency conditions [36].

It is now well established that PA is connected to autophagy, although the exact roles played by PA are not yet clear and in some cases are contradictory [37–39]. The present findings showed that exogenous PA treatment alone did not stimulate or suppress autophagy. However, the results of combination treatment of both PA and α-mangostin showed that α-mangostin induced autophagy could be significantly reversed by exogenous PA, indicating that the fatty acid reduction may be one of the reasons for α-mangostin induced autophagy.

In addition to its ability to promote cancer by allowing cells to survive under conditions of nutrient deficiency, autophagy may paradoxically lead to cell death [40]. We next investigated the role of autophagy in α-mangostin induced apoptosis by treatment with 3MA. We found that 3MA itself in the absence of α-mangostin was not toxic. The combination of 3MA and α-mangostin reduced cell viability significantly and enhanced α-mangostin induced cell apoptosis, which clearly demonstrated a potent cooperative effect of FAS inhibition and autophagy inhibition.

As one of the largest organelles observed in eukaryotes, ER facilitates folding, maturation, in addition to the synthesis of secreted and transmembrane cellular proteins under homeostatic conditions. However, cellular disturbances triggered by changes in physiological or environmental factors can elicit the ER to experience ER stress [41]. To explore whether FAS inhibition was one of the reasons for α-mangostin inducing ER stress in MDA-MB-231 cells, PA, the end product of FAS catalyzed reaction, was applied with and without α-mangostin treatment. The results showed that PA itself did not trigger ER stress. However, compared with α-mangostin treatment alone, the combination treatment of both PA and α-mangostin suppressed ER stress significantly, indicating that FAS inhibition triggers ER stress.

ER stress response is crucial for cancer cells survival. On the other hand, prolonged ER stress and activation of UPR pathways can lead to apoptosis and autophagy [42]. The role of ER stress in α-mangostin induced apoptosis was investigated by treatment with 4PBA. 4PBA treatment alone showed no toxic on both MDA-MB-231 and MCF-7 cells. However, 4PBA markedly enhanced α-mangostin induced apoptosis as well as decreased cell viability, suggesting that ER stress may serve a protective role in α-mangostin induced apoptosis in breast cancer cells.

As mentioned above, both autophagy and ER stress are responses to stress in cancer cells. Moreover, there is often the interplay between these responses that ultimately determines the fate of the stressed cancer cell. Previous studies demonstrated that ER stress is an autophagy inducer [43–45]. Consistent with this, our results also indicate that ER stress is involved in α-mangostin induced autophagy, cause ER stress inhibitor rescued autophagy. Interestingly, we found that autophagy inhibitor depressed ER stress also. Cross-talk between ER stress and autophagy is evident from our findings that ER stress inhibition reduced autophagy and autophagy inhibition reduced ER stress. Based on the time course results (Additional file 2: Figure S2), the expression levels of both ER stress and autophagy markers were up-regulated within 12 h. The expression levels of P62, BIP, LC3II/LC3I were up-regulated with a time-dependent manner. However, the expression level of CHOP was up-regulated significantly in 6 h, and then was down-regulated after 12 h. The relationship between ER stress and autophagy is still not clear from these results.

It is now generally accepted that both ER stress and autophagy participate in cell death under some circumstances and promote cell survival under others. Our results showed that the apoptosis induced by α-mangostin could be restored by autophagy or ER stress inhibitor. It is strongly suggested that autophagy and ER stress had a protective effect on α-mangostin induced breast cancer cell death. Considering that both 3MA and 4PBA did not affect cell viability and apoptosis, the protective effects of them on α-mangostin induced cell apoptosis may not be due to their direct effects on breast cancer cells. From the above results we can draw a conclusion that autophagy and ER stress inhibition enhanced the cytotoxicity of α-mangostin.

**Fig. 7:**
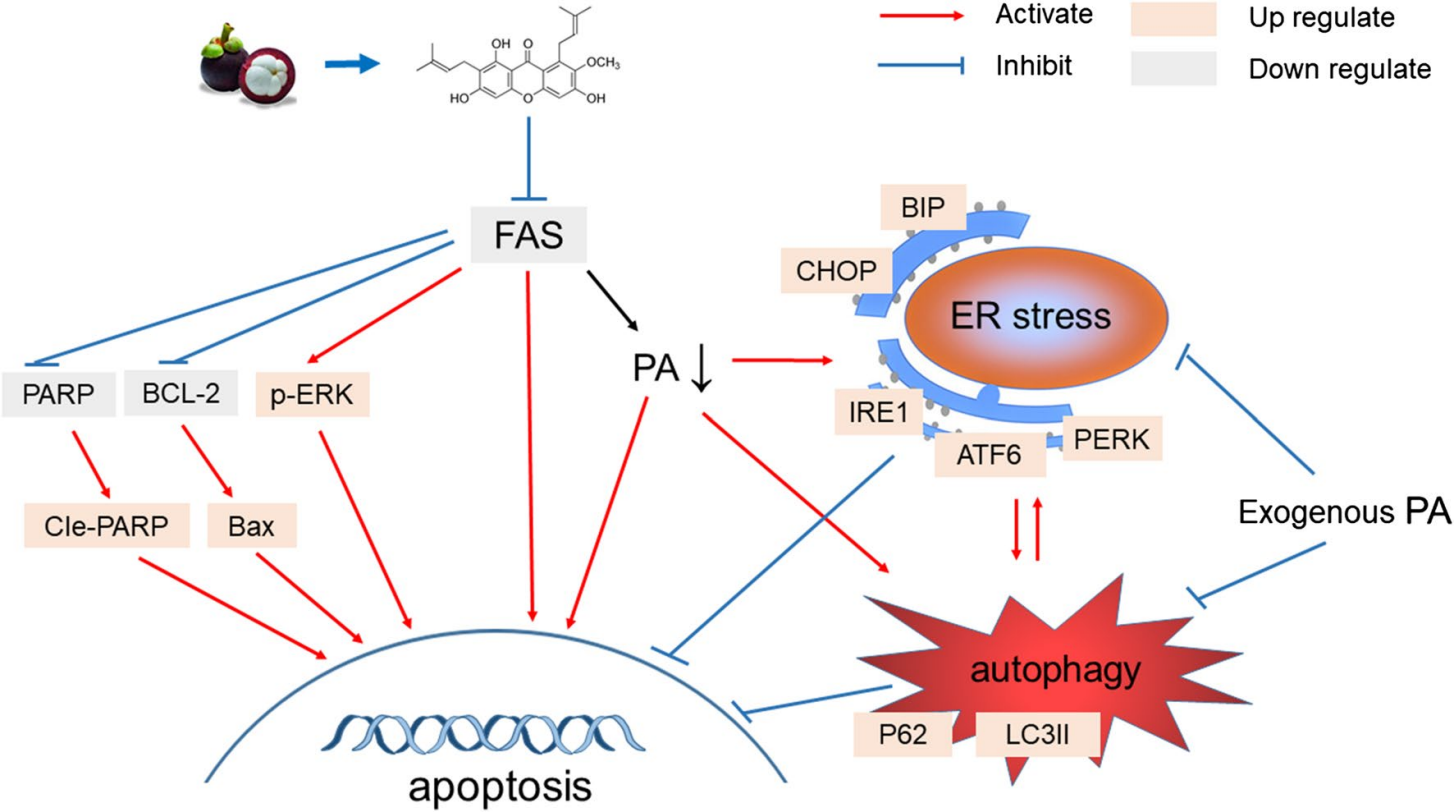
The mechanism of α-mangostin induced apoptosis

Fatty acid synthesis has now become a new target for the cancer treatment. In addition, it is known that the decrease in activity or expression of FAS can effectively reduce cancer growth or induce apoptosis. However, the reduction of fatty acid by FAS inhibition may trigger some extra protection to maintain the cell survivals. This study has evaluated, for the first time, the influence of FAS inhibition on ER stress, autophagy, and further synergetic or antagonistic effect on cancer cell apoptosis. FAS inhibition could induce both ER stress and autophagy, which in turn become obstacles that suppress the apoptosis activity of FAS inhibitor (Fig. 7). Therefore, we conclude that a combine inhibition of FAS, ER stress, and autophagy has a therapeutic potential, giving a novel means of controlling breast cancer. Some potential limitations of this study should be considered. All the results in this study were based on cell experiments. However, the effect of autophagy inhibitor, ER stress inhibitor, and α-mangostin on animals is not clear and needs to be clarified in vivo.

## Conclusions

In summary, this study suggests that autophagy and ER stress, both of which can be reversed by exogenous PA, are involved in the mechanism of α-mangostin resistance in human breast cancer cells. Combined with their negative interaction, we provide the basis for future preclinical and clinical trials exploring co-inhibitors of FAS, autophagy and ER stress as a combinatory therapeutic approach for human breast cancer.

## Additional files

**Additional file 1: Figure S1.** The effects of α-mangostin on ER stress, autophagy, cell viabilities in MCF-7 cells. (A) MCF-7 cells were treated with 0, 1, 2, and 4 μM α-mangostin for 24 h, and then the relative expression levels of CHOP, BIP, LC3II/LC31 and P62 were analyzed by western blot and were quantified densitometrically with the software ImageJ and calculated according to the reference bands of GAPDH. Data represented the mean ± SD of three independent experiments. **p < 0.01. (B) MCF-7 cells were treated with 4 μm α-mangostin, 5 mM 4PBA, 5 mM 3MA or a combination of them. Cell viabilities were then determined by the CCK-8 assay. Data represented the mean ± SD of three independent experiments. **p < 0.01. (C) MCF-7 cells were treated with/without 4 μm α-mangostin followed 24 h incubation with/without 10 μM PA. Cell viabilities were then determined by the CCK-8 assay. Data represented the mean ± SD of three independent experiments. **p < 0.01.

**Additional file 2: Figure S2.** The time-dependent effects of α-mangostin on ER stress and autophagy in MDA-MB-231 cells. Cells were treated with 4 μm α-mangostin for 0, 6, 12, 18, and 24 h, and then the relative expression levels of CHOP, BIP, LC3II/LC31 and P62 were analyzed by western blot and were quantified densitometrically with the software ImageJ and calculated according to the reference bands of GAPDH. Data represented the mean ± SD of three independent experiments. *p < 0.05, **p < 0.01.

**Additional file 3: Figure S3.** α-Mangostin inhibited intracellular FAS activity and reduced the amount of free fatty acids. (A) MDA-MB-231 cells were treated with 0, 1, 2, and 4 μM α-mangostin for 24 h, then intracellular FAS activity was determined spectrophotometrically by measuring the decrease of absorbance at 340 nm due to oxidation of NADPH. (B) MDA-MB-231 cells were treated with 0, 1, 2, and μM α-mangostin for 24 h. Then cells were harvested using trypsin–EDTA, washed twice with PBS. Intracellular fatty acid was determined with a Free Fatty Acid Quantification Kit (Bivision) according to the manufacturer’s instructions. Data represented the mean ± SD of three independent experiments. *p < 0.05, **p < 0.01.

## Data Availability

All data analyzed and generated during the current study are available from the corresponding author upon reasonable request.

## Abbreviations

3MA: 3-methyladenine
4PBA: 4-phenylbutyrate
ATF6: activating transcription factor 6
CCK-8: Cell Counting Kit
CoA: coenzyme A
DMEM: Dulbecco’s modified Eagle’s medium
ER: endoplasmic reticulum
FAS: fatty acid synthase
FBS: fetal bovine serum
IC_50_: the half inhibition concentration
IRE1: inositol-requiring enzyme
MDC: monodansylcadaverine
PA: palmitic acid
PBS: phosphate buffer saline
PERK: protein kinase R-like endoplasmic reticulum kinase
UPR: unfold protein response
