# Supplementary figures and images for: Alpha-mangostin induces endoplasmic reticulum stress and autophagy which count against fatty acid synthase inhibition mediated apoptosis in human breast cancer cells
Source: Cancer Cell Int. 2019 May 31;19:151. doi: 10.1186/s12935-019-0869-z (PMC6544980; doi:10.1186/s12935-019-0869-z)

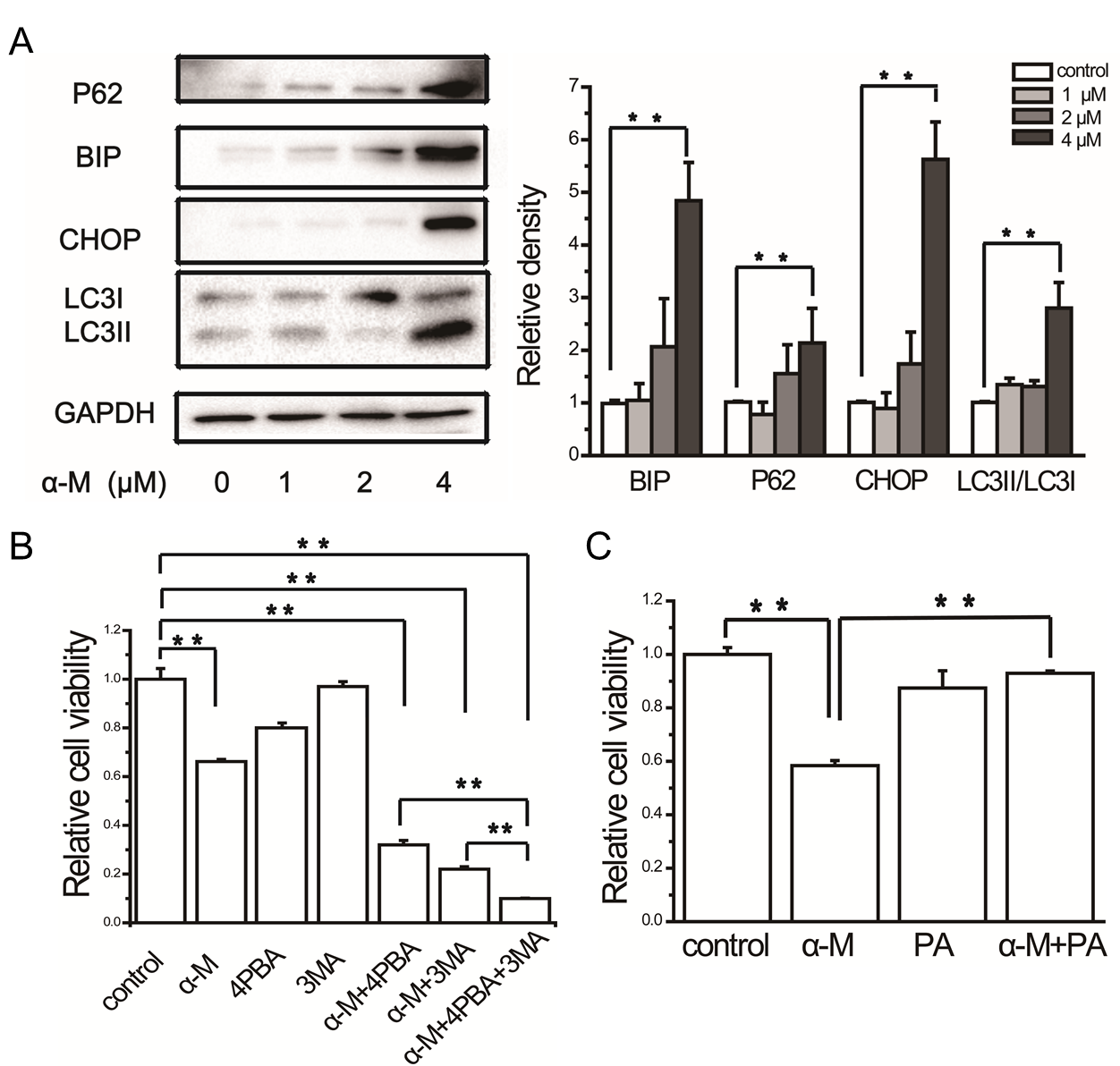

Supplement: Supplementary file 1 — Additional file 1: Figure S1. The effects of α-mangostin on ER stress, autophagy, cell viabilities in MCF-7 cells. (A) MCF-7 cells were treated with 0, 1, 2, and 4 μM α-mangostin for 24 h, and then the relative expression levels of CHOP, BIP, LC3II/LC31 and P62 were analyzed by western blot and were quantified densitometrically with the software ImageJ and calculated according to the reference bands of GAPDH. Data represented the mean ± SD of three independent experiments. **p < 0.01. (B) MCF-7 cells were treated with 4 μm α-mangostin, 5 mM 4PBA, 5 mM 3MA or a combination of them. Cell viabilities were then determined by the CCK-8 assay. Data represented the mean ± SD of three independent experiments. **p < 0.01. (C) MCF-7 cells were treated with/without 4 μm α-mangostin followed 24 h incubation with/without 10 μM PA. Cell viabilities were then determined by the CCK-8 assay. Data represented the mean ± SD of three independent experiments. **p < 0.01. [file 12935_2019_869_MOESM1_ESM.tif]

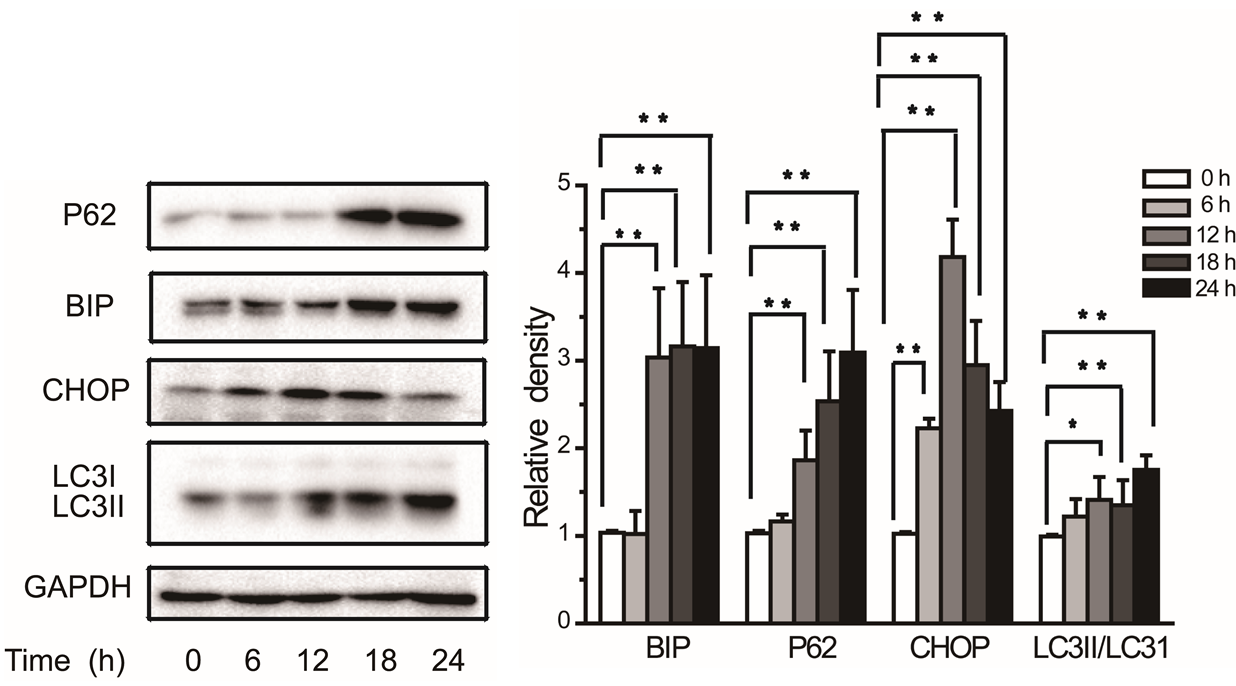

Supplement: Supplementary file 2 — Additional file 2: Figure S2. The time-dependent effects of α-mangostin on ER stress and autophagy in MDA-MB-231 cells. Cells were treated with 4 μm α-mangostin for 0, 6, 12, 18, and 24 h, and then the relative expression levels of CHOP, BIP, LC3II/LC31 and P62 were analyzed by western blot and were quantified densitometrically with the software ImageJ and calculated according to the reference bands of GAPDH. Data represented the mean ± SD of three independent experiments. *p < 0.05, **p < 0.01. [file 12935_2019_869_MOESM2_ESM.tif]

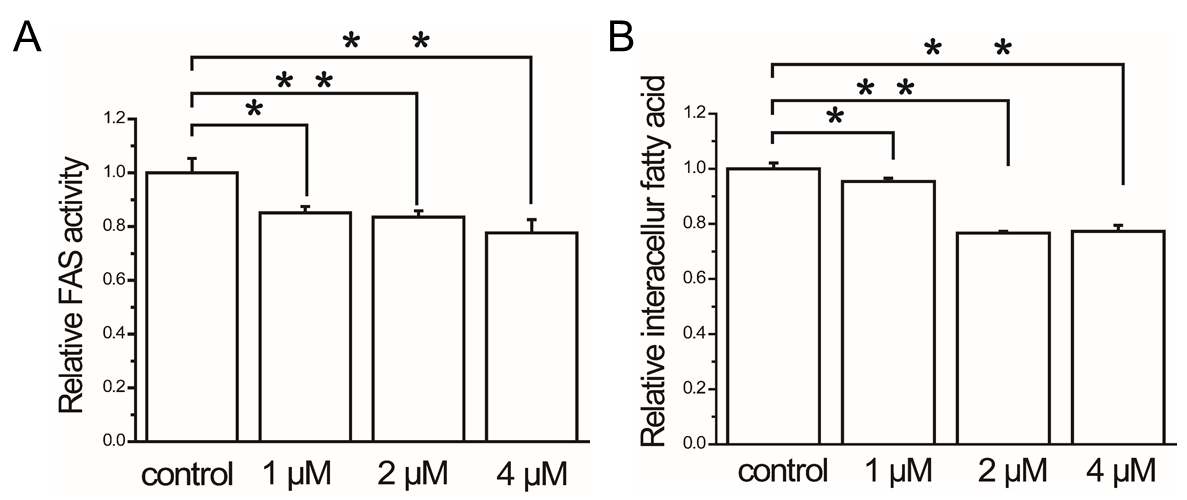

Supplement: Supplementary file 3 — Additional file 3: Figure S3. α-Mangostin inhibited intracellular FAS activity and reduced the amount of free fatty acids. (A) MDA-MB-231 cells were treated with 0, 1, 2, and 4 μM α-mangostin for 24 h, then intracellular FAS activity was determined spectrophotometrically by measuring the decrease of absorbance at 340 nm due to oxidation of NADPH. (B) MDA-MB-231 cells were treated with 0, 1, 2, and μM α-mangostin for 24 h. Then cells were harvested using trypsin–EDTA, washed twice with PBS. Intracellular fatty acid was determined with a Free Fatty Acid Quantification Kit (Bivision) according to the manufacturer’s instructions. Data represented the mean ± SD of three independent experiments. *p < 0.05, **p < 0.01. [file 12935_2019_869_MOESM3_ESM.tif]
